# Identifying Somatic Lymph Nodes for VLNT Using Technetium-99m Sulfur Colloid

**DOI:** 10.1055/s-0045-1811168

**Published:** 2025-09-23

**Authors:** Srikanth Vasudevan, Anantheswar N. Yelambalase, Ashok Basur Chandrappa, Mayur R. Shetty, Parameswaran RV, Annika Marwah, Pooja Aditya Shetty, Serena K. Bharathkar, Aashita R. Yande, Chinmay Tewari, Ria Pai, Milind Sharma, Nitisha Sara

**Affiliations:** 1 Department of Plastic Surgery, Manipal Hospitals, Bengaluru, Karnataka, India; 2 Department of Plasic Surgery, Aster Whitefield Hospital, Bengaluru, Karnataka, India; 3 Department of Nuclear Medicine, Manipal Hospitals, Bengaluru, Karnataka, India

**Keywords:** Technetium-99m, vascularized lymph node, lymphoedema, localizing lymph nodes

## Abstract

Vascularized lymph node transplant (VLNT) is one of the treatment options for chronic lymphedema following both breast cancer-related lymphoedema and lower extremity lymphoedema. VLNT is a safe and effective treatment for lymphedema with significant benefits fully manifesting at 2 years postoperatively. This involves the microvascular transfer of lymph nodes to the affected limb. The transferred lymph nodes then act as a sump to drain the excessive lymphatic fluid in the interstitial space. One of the challenges in VLNT is to include an adequate number of lymph nodes in the flap, which requires some way of identifying them before harvesting. In order to transfer lymph nodes along with their vascularity, we have relied on anatomic studies. However, using Technetium-99m sulfur colloid as used in sentinel lymph node harvest, we can identify lymph nodes in the transferred tissue, giving greater reliability to the procedure. It involves identifying the lymph node area before incision, guiding surgerons during harvest of the lymph nodes along with the vascularity, confirming the presence of lymph nodes after harvest, and confirming the presence after microvascular transfer to the affected site. It can be used along with methylene blue dye and indocyanine green (ICG) to confirm the presence of lymph nodes. In our pilot study of eight cases, we have found the presence of lymph nodes in all the transferred tissue. This is in comparison to certain studies on sentinel lymph node studies that indicate that the use of radiotracer and ICG is comparable in localizing lymph nodes.

## Introduction

Lymphedema is a common, debilitating, and often misunderstood disease. Vascularized lymph node transplant (VLNT) provides satisfactory reduction in limb volume, bioimpedance, and improved quality of life. Transferring vascularized lymph nodes to the affected limb is one solution. The primary treatment is lifelong compression and manual lymphatic drainage. While these therapies are essential for managing swelling, they do not treat the underlying disease. The proposed mechanism for VLNT is that the lymph entering the transplanted lymph nodes is shunted into the venous system via interconnections between the lymphatic sinuses and venules within the transplanted nodes. 1 We have been using various donors of VLNT such as omental, right supraclavicular, groin, and chest wall lymph nodes. In all these methods, actual identification of the lymph nodes has always been a challenge. 2 The use of radiotracers such as Technetium-99m (Tc-99m) has helped us confirm the presence of lymph nodes in all our VLNT procedures.

## Materials and Methods

**Fig. 1:**
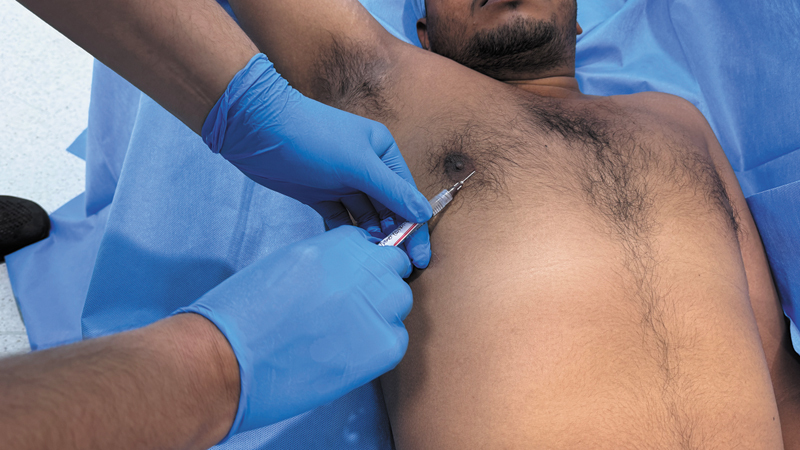
Photo of injection subareolar dye.

Tc-99m, a radiotracer dye, was used in identifying the lateral thoracic wall lymph nodes. 3 4 This is the same dye used in identifying sentinel lymph nodes in breast conservation surgery. 2 5 The technique is as follows. A subareolar injection of 1 mL of Tc-99m is given about 2 hours prior to the surgery ( Fig. 1 ). After the patient is under general anesthesia, a gamma probe is used to locate the lymph nodes. A gamma probe count of 10% of the highest count and above is considered a positive finding. 6 Our studies have shown a variation between 25 and 450 counts on the meter. When performed with proper technique and training and taking appropriate precautions, the donor-site morbidity of lateral thoracic node harvest is not greater than that of other donor sites. We use the contralateral chest wall lymph nodes as the donor site. The lateral thoracic artery, which is the classic pedicle to the lateral thoracic lymph nodes, originates from the axillary artery, but anatomic variations can exist, with variable origin or complete absence of the lateral thoracic artery altogether. An alternative pedicle is the thoracodorsal artery, which can also supply the lateral thoracic nodes. We do not use a skin paddle along with the nodes. 3 The incision is made along the anterior axillary line, and the dissection proceeds posteriorly over the anterior border of the latissimus dorsi muscle. The lateral thoracic nodes are lateral to the breast and pectoralis major muscle and can extend deeply between the muscle and the chest wall. On average, three nodes in this location can be transferred safely based on either the lateral thoracic or thoracodorsal pedicle. The lateral thoracic pedicle is often shorter than the thoracodorsal pedicle, but has a usable average diameter of 2.2 mm and average length of 3.6 cm. The thoracodorsal pedicle can be considerably longer with a larger caliber artery and vein.

**Fig. 2:**
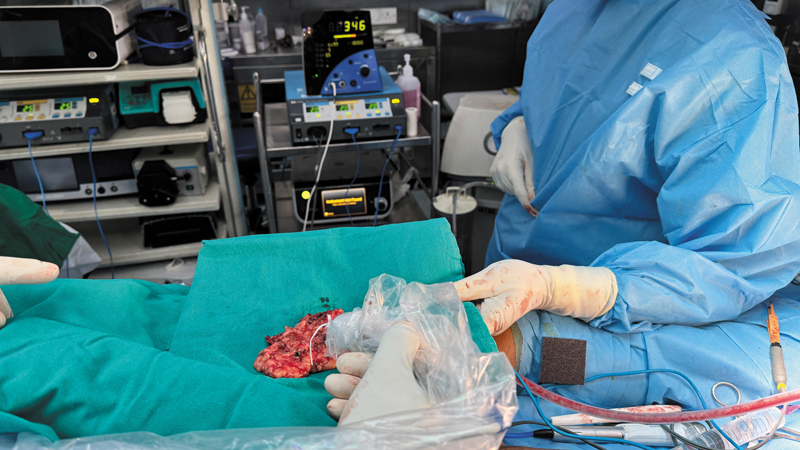
Ex vivo photo of flap and count on monitor.

**Fig. 3:**
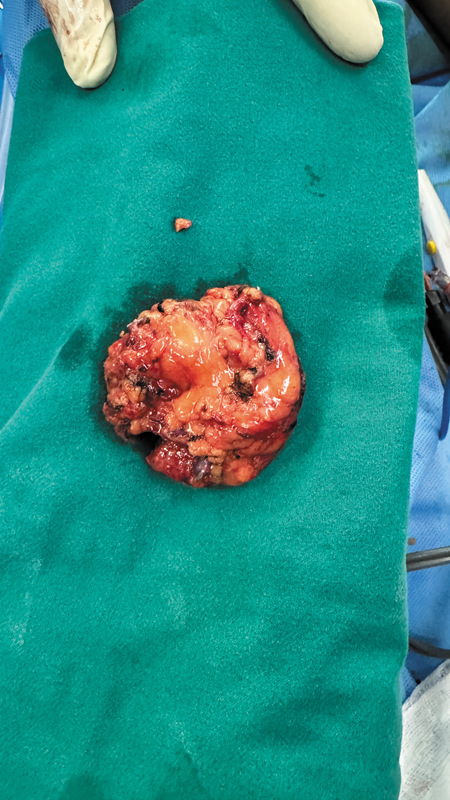
Flap ex vivo.

The long thoracic nerve is always spared. The vascularity is confirmed by indocyanine green (ICG), and the presence of the lymph node is confirmed using a gamma camera. Typically, we have found a count of 100 and above. This is considered a positive finding for a lymph node ( Fig. 2 ). 6 The mass of lymph nodes, along with the soft tissue, is harvested on the lateral thoracic vessels or the thoracodorsal vessels ( Fig. 3 ).

**Fig. 4:**
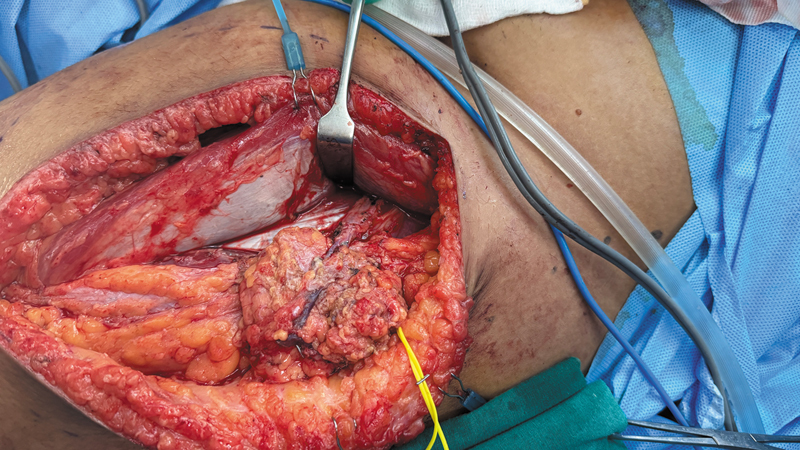
Intraoperative photo of flap and count on monitor.

**Fig. 5:**
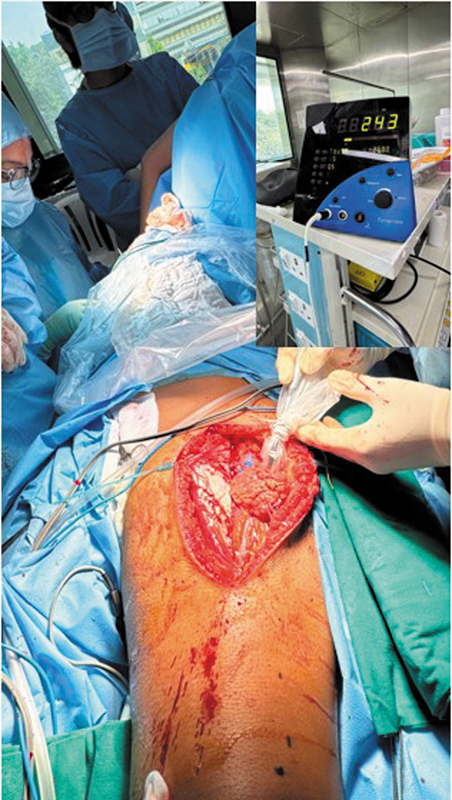
Post-anastomosis flap and count on monitor.

The flap is then transferred to the recipient site, and microvascular anastomoses are done. Usually, a subcutaneous pocket is cored out to accommodate the flap. The vascularity of the flap is confirmed using ICG ( Fig. 4 ). The presence of the lymph nodes can be confirmed by the gamma probe ( Fig. 5 ).

The flap donor site is closed over a drain. The drain is removed on days 3 to 5 when the drain is approximately 15 to 20 mL. The presence of lymph nodes is confirmed using the gamma camera at the site of transfer.

Postoperative care of the free flap includes Doppler monitoring, drain monitoring, administration of low-molecular-weight heparin, and intravenous fluids for about 5 days.

## Discussion

VLNT is gaining traction for the treatment of mild to moderate lymphoedema. The donor sites are many. We have used the supraclavicular, omental, lateral thoracic wall, and groin in various instances. Of the choices, we have found the use of lateral chest wall nodes to be convenient. Conceptually, the transfer of a somatic set of lymph nodes seems better than a visceral set of lymph nodes. When compared to other donor sites, the advantages are many. No need to involve another surgeon (lowering costs), reluctance of the patients to undergo a laparoscopic procedure, predictable anatomy, reliable blood vessels, etc., are a few that made us gravitate toward the thoracic wall lymph nodes as a donor site. There is often uncertainty regarding the presence and number of lymph nodes during transfer. By using Tc99m radiotracer, we have ascertained the presence and number of lymph nodes prior to, during and after vascularised lymph node transfer.

We have used this technique in eight cases. In all eight cases, we could identify the radiotracer dye in the region where the lymph nodes were expected to be. The number ranged from 3 to 5 ( Supplementary Table , available in the online version). In about three cases, methylene blue also helped identify the nodes in the expected region. ICG did help only partially, as it was difficult to isolate only the lymph nodes. By the time the tissue was harvested, we could see that the mass of the flap was lit up in the camera due to contamination at the site. We prefer to use the ICG for confirming flap vascularity.

As compared to radiotracer, other methods, such as ICG and methylene blue, have a few challenges in confirming the presence of lymph nodes in the flap 7 8 9 ( Table 1 ). However, we have found the ICG to be difficult to interpret as the flap with nodes needs to be harvested quickly before it gets washed out or gets diffused. Methylene blue is not too reliable. We have found that lymph nodes can be identified with a faint green tinge in about 30% of the cases. The use of the radiotracer has helped us identify the lymph nodes preoperatively, before transfer, and after transfer, thus increasing the confidence that the VLNT will work well ( Figs. 1 , 2 , 3 , 4 , 5 ).

**Table 1:** Comparing salient features of the dyes used in sentinel lymph node biopsy

Dye	Instrument cost	Dye cost	Time of injection	Sensitivity	Specificity	On table contamination
Technetium-99m	13 lakhs	1,000	2 h prior	90–92%	100%	Nil
Indocyanine green	22 lakhs	2,000	At induction	71%	85–96%	Yes
Methylene blue	Nil	330	1/2 h prior	87–94%	86–100%	Nil

## Conclusion

VLNT is gaining traction in the treatment of both upper limb and lower limb lymphedema. There are many sites for the harvest of vascularized lymph nodes. Of all donor sites, omental lymph nodes have gained popularity in the recent past. This, however, necessitates the need to convince the patient of the need for an abdominal procedure, a laparoscopic surgeon, which adds to the cost and coordination challenges regularly. Conceptually, we feel somatic lymph nodes should work better. The use of chest wall lymph nodes has declined in popularity. Our experience with chest wall lymph nodes has shown that we are able to harvest adequate lymph nodes from the chest wall with a predictable vascular pedicle. The use of Tc-99m radiotracer has helped identify the presence of lymph nodes preoperatively, intraoperatively, and posttransfer reliably. We recommend this technique to add to the armamentarium of surgeons involved in the treatment of lymphoedema.
